# Analysis of writing in personality disorders in prison population

**DOI:** 10.3389/fpsyt.2024.1391463

**Published:** 2024-05-24

**Authors:** Lucas Muñoz-López, Borja Fernández-García-Valdecasas, Slava López-Rodríguez, María Blanca Sánchez-Barrera

**Affiliations:** ^1^ Department of Personality, Evaluation and Psychological Treatment, Faculty of Education and Sports Sciences, University of Granada, Melilla, Spain; ^2^ Department of Theory and History of Education, Faculty of Education Sciences, University of Granada, Granada, Spain; ^3^ Department of Didactics of Language and Literature, University of Granada, Granada, Spain; ^4^ Department of Personality, Evaluation and Psychological Treatment, Faculty of Psychology, University of Granada, Granada, Spain

**Keywords:** writing disabilities, impulsive-compulsive, drugs possession/consumption, gender violence, PROESC

## Abstract

**Abstract:**

Writing involves the activation of different processing modes than reading comprehension, and therefore the level of activation varies depending on the moment and the task.

**Objectives:**

to analyze the profiles in terms of the proposed coding from the PROESC in terms of personality disorders [Antisocial Personality Disorder (ASPD) with drugs possession and consumption crimes (DPCC) and Obsessive-Compulsive Personality Disorder (OCPD)] with gender violence crimes (GVC) in the prisoners.

**Design:**

The sample was composed of 194 men. The participants were divided into two groups. Group 1 (ASPD; DPCC) consisted of 81 men, and Group 2 (OCPD; GVC) consisted of 113 men.

**Main outcome measures:**

They completed the Demographic, Offense, and Behavioral Interview in Institutions, the International Personality Disorders Examination (IPDE), and Writing Processes Evaluation Battery (PROESC).

**Results:**

Group 2 made more mistake than Group 1 in narratives tasks.

**Conclusion:**

Participants know phoneme-grapheme correspondence rules, language disturbances of a reiterative and persistent nature may appear in those who show compulsive behavior.

## Introduction

Aggression can be conceptualized as impulsive or compulsive behavior, and depending on whether it is impulsive or compulsive, the treatment will be different. Impulsive behaviors can often be controlled, whereas compulsive behaviors require more specialized and multifactorial treatment (neuropsychological, neurophysiological, neuroanatomical, social, legal, safety, and economic), as they are usually part of a more severe problem (1, 2). Impulsivity and compulsivity are natural behaviors driven by brain mechanisms that are essential for survival in all species. Understanding these brain mechanisms can lead to targeted treatment strategies for these symptom areas when impulsivity and compulsivity become dysfunctional (3). Although impulsivity and compulsivity affect different aspects of response control, they are most likely mediated by related but distinct neural circuits associated with motivational and decision-making processes (involving the basal ganglia, their limbic cortical inputs, and top-down control by cortical prefrontal circuits) (1, 4). Ziegler et al. (2) argued that increased frontal lobe activity may characterize obsessive-compulsive disorders such as OCD. In contrast, decreased frontal lobe activity may characterize impulsive disorders such as substance abuse (SAD) and antisocial personality disorder (ASPD). In addition, numerous studies (1, 5) support the association between impulsivity, substance abuse disorders (SAD) and violent or aggressive behavior. In contrast, according to some authors (3, 4, 6), research linking impulsivity to such outcomes is sparse, as impulsivity has often been studied in OCD. It is then necessary to examine impulsivity and compulsivity from the same group of disorders in the DSM-5 (7). Therefore, it is important to distinguish writing disorders between these two disorders.

Writing involves the activation of different processing modes than reading comprehension, and therefore the level of activation varies depending on the moment and the task.

Information processing comprises a series of stages or sub-processes in mental operations that may act in a more or less autonomous and task-specific manner. Consequently, explicit responses are the result of these operations, and thus language assessment is concerned with identifying these processes to determine correct functioning. In this regard, the goal of language assessment is to discover the sequence of information processing that takes place from the time the individual receives it until it is manifest in an explicit response. Evaluating a given process (in this case, writing) implies knowing the transformations of the written text, from the activation of a mental representation as an abstract schema to the creation process (8–20).

The Writing Processes Evaluation Battery (PROESC) (21) is an individual test that aims to evaluate the main processes involved in creating texts. It is composed of six tests, which are: 1) Syllable dictation; 2) Word dictation; 3) Pseudoword dictation; 4) Sentence dictation; 5) Writing a narrative and 6) Writing an essay. Tests 5 and 6 assess the ability to plan a narrative text and an expository text and involve qualitative aspects.

Currently, there are many standardized tests to assess language and detect language difficulties in adults in opaque languages such as English. In other languages, such as Spanish, there are not many options. Moreover, a review study (22) concluded that research on language ability and language disorders has mainly focused on children and adolescents. Studies on the adult population are scarce and, to a large extent, adopt the perspective of adults who were diagnosed with disorders as children. However, these studies have developed methods for identifying, for the first time, language development deficits in English-speaking adults. Therefore, the objective of this study was to analyze the profiles based on the proposed coding using the PROESC (21) in terms of personality disorders (ASPD and OCPD) in the prison population.

## Participants

The sample consisted of 194 men with a mean age of 37.08 years (SD=8.81) from the Granada Penitentiary Center. The participants were divided into two groups. Group 1 presented antisocial personality disorder (ASPD), composed of 81 men with a mean age of 36.86 years (SD=9.32), while Group 2 presented Obsessive-Compulsive Personality Disorder (OCPD) and was composed of 113 men with a mean age of 38.78 years (SD=8.47). The exclusion criteria in both cases were being over 50 years, presenting a psychiatric illness (schizophrenia or depression), and receiving psychopharmacological treatment. **Table 1** present the sociodemographic characteristics of the sample described.

**Table 1:** Sociodemographic Variables of the sample analyzed.

	N=194			
	Group APD	Group OCPD	χ^2^	*p*
**Marital status (N)**			**10.916**	**0.028**
Single	41	48		
Married	10	35		
Divorced	12	15		
Widower	1	0		
Cohabiting with partner	17	15		
**Educational level (N)**			1.575	0.813
No Primary	17	16		
Primary	33	51		
Secondary	21	31		
Baccalaureate	8	12		
Degree	2	3		
**Nationality (N)**			1.558	0.669
Spain	78	106		
Europe	0	2		
South America	2	3		
Africa	1	2		

Bold values in the tables represent those values that are statistically significant (p ≤ 0,05).

## Procedure

First, participants were interviewed individually to check the inclusion criteria and, if eligible, were offered the opportunity to participate in the research. Next, they completed the International Personality Disorders Examination (IPDE) (23), and participants with Antisocial Personality Disorder (ASPD) and Obsessive-Compulsive Personality Disorder (OCPD) were selected. They then took part in an individual session in which they completed the measures listed below. Participants were reminded at the beginning of the session of their right to discontinue the procedure at any time, and their written consent was then obtained. Once the data collection process was completed, the data were corrected.

Finally, participants signed the informed consent form, and prison staff (psychologist and educator) collected the relevant sociodemographic data. This study was approved by the Ethics Committee of the Autonomous Community of Andalusia (PEIBA, 0766-N-21).

## Instruments

### Demographic, crime, and institutional behavior interview

This interview was designed for this research study and consists of collecting information about sociodemographic data, type of offenses and their penalties, and sanctions within the prison according to the Prison Regulations (Royal Decree 1201/1981, May 8, Articles 107 and 108).

### International personality disorders examination (IPDE) Spanish version

This is a diagnostic instrument based on a semi-structured clinical interview (23), and his Spanish version (24), formulated according to the DSM-5 (7) assessment criteria. The items are open-ended, closed-ended, and yes/no questions are classified into six categories: work, self, interpersonal relationships, affect, reality testing, and impulse control. The instrument also includes a screening questionnaire that reduces the interview administration time, identifying personality disorders in which the person does not score and, therefore, discarding the questions referring to that disorder. The administration time ranges from 60 to 90 minutes and requires an examiner with training and experience in using the instrument. The reliability and stability indices obtained range between 0.70 and 0.96. It has been considered a useful and valid instrument for assessing personality disorders for research purposes (23).

### Writing processes evaluation battery (PROESC)

This is an individual test that aims to evaluate the main processes involved in creating texts (21). It is composed of six tests, which are: 1) Syllable dictation; 2) Word dictation; 3) Pseudoword dictation; 4) Sentence dictation; 5) Writing a narrative and 6) Writing an essay. In this study, we used tests 5 and 6, which assess the ability to plan a narrative and an expository text. Although the instrument (21) has a high internal consistency of 0.82 (alpha coefficient) in the first four tests, it lacks quantitative criteria for the correction and interpretation of the writing tests (5 and 6). Our proposal of criteria for correction and interpretation was: Words and Paragraphs, Errors Related to Formal Aspects, Decoding Errors, Grammar, Revision and Net Total, Main and Secondary Ideas, Vocabulary, Planning Errors, Words and Paragraphs, Errors Related to Formal Aspects, Decoding Errors.

### Data analysis

Data analyses were conducted using the SPSS Statistics 22.0 program. First, descriptive statistical analyses were used to determine the characteristics of the sample. Then, to analyze the profiles around the proposed coding from the PROESC (25) to categorize the *narratives* and *essays* according to personality disorders (ASPD and OCPD), we proceeded to check whether the narratives obtained according to the PROESC instructions differed between the groups. For this purpose, seven Multivariate analyses of Variance (MANCOVA) were conducted using a between-groups unifactorial design, using educational level as a covariate; the group (ASPD and OCPD) as the independent variable, and the variables derived from the categories (Category Words and Paragraphs; Errors Related to Formal Aspects; Decoding Errors; Category Grammar/Revision/Net Total; Main and Secondary Ideas; Planning and Vocabulary Errors) as dependent variables.

## Results

### Analysis of narrative categorization

The MANCOVAS revealed statistically significant results for the categories *Words and Paragraph* **s** (Number of words and Number of paragraphs; Wilks’ Lambda = 0.933, F_2,190_ = 6.866; p <.01); *Errors Related to Formal Aspects* (Number of punctuation errors, number of lines not respecting the margins, number of incorrect separations between words, number of incorrect conjunctions between words, number of repetitions, number of words with unreadable handwriting, and Total; Wilks’ Lambda = 0.74, F_6,186_ = 10.912; p <.001); *Decoding Errors* (Number of Substitutions, number of Additions, number of Omissions, number of Inversions, number of Rotations, number of Lexicalizations, Number of incorrect accents and Total; Wilks’ Lambda = 0.686, F_7,185_ = 12.107; p <.000); *Grammar* (Number of grammatically incorrect sentences); *Revision* (Number of modifications made to the text) and *Net Total*; Wilks’ Lambda = 0.801, F_3,189_ = 15.699; p <.001); *Main and Secondary Ideas* (Number of main ideas and number of secondary ideas; Wilks’ Lambda = 0.646, F_2,190_ = 52.032; p <.001); *Vocabulary* (Number of technical vocabulary uses, number of coherent vocabulary uses, number of varied vocabulary uses and Total; Wilks’ Lambda = 0.94, F_3,189_ = 3.998; p <.01). No statistically significant differences were found in the *Planning Errors* category (number of disconnections between the main idea and the title, number of times secondary ideas do not appear, number of deviations from thematic continuity, number of times technical vocabulary is not used, number of times coherent vocabulary is not used, number of times varied vocabulary is not used, and Total).

Univariate ANCOVAs conducted for each of the levels of the dependent variables of the *Words and Paragraphs* category (Number of Words and number of Paragraphs) revealed statistically significant differences in the number of words (F_2,191_ = 10.150; Mce =5684.14; p<.001) with the scores being higher for the OCPD group than the ASPD group; and in the number of paragraphs (F_2,191_ = 13.76; Mce =21.75; p<.001) with the scores being higher for the OCPD group than the ASPD group (See **Table 2**).

**Table 2:** Mean. standard deviation. and significance level obtained by the groups when comparing.

		NARRATIVES							ESSAYS						
CATEGORIES	VARIABLES	Group APD		Group OCPD		F/χ^2^	*p*	*η*	Group APD		Group OCPD		F/χ^2^	*p*	*η*
		Mean	SD	Mean	SD				Mean	SD	Mean	SD			
WORDS AND PARAGRAPHS	Number of words	**139**	**66.74**	**156**	**85.20**	**10.150**	**0.000**	**0.096**	**136.83**	**67.54**	**151.26**	**69.60**	**6.63**	**0.002**	**0.065**
	Number of paragraphs	**1.6**	**1.05**	**1.9**	**1.51**	**13.758**	**0.000**	**0.126**	**1.65**	**1.15**	**1.71**	**1.10**	**12.475**	**0.00**	**0.116**
ERRORS RELATING TO FORMAL ASPECTS	Number of punctuation errors	6.53	4.37	7.25	5.30	1.955	0.144	0.020	**7**	**5.10**	**7.24**	**4.56**	**3.337**	**0.038**	**0.034**
	Number of lines not respecting margins	0.60	2.63	0.79	2.74	0.215	0.806	0.002	1.05	3.38	0.59	2.12	1.386	0.253	0.014
	Number of incorrect separations between words	0.31	0.79	0.49	1.56	1.731	0.180	0.018	0.26	0.70	0.34	1	0.872	0.420	0.009
	Number of incorrect conjunctions between words	**0.42**	**1.04**	**0.73**	**2.26**	**4.558**	**0.012**	**0.046**	0.78	1.80	0.72	1.67	2.457	0.088	0.025
	Number of repetitions	0.01	0.11	0.02	0.13	0.055	0.946	0.001	0	0	0.02	0.13	0.733	0.482	0.008
	Number of words with unreadable handwriting	0.26	0.79	0.38	1.37	1.797	0.169	0.018	0.14	0.38	0.42	1.75	2.580	0.078	0.026
	TOTAL	**8.14**	**5.65**	**9.65**	**8.04**	**3.723**	**0.026**	**0.038**	**9.22**	**6.74**	**9.32**	**6.44**	**6.238**	**0.002**	**0.061**
DECODING ERRORS	Number of Substitutions	**4.67**	**3.74**	**4.70**	**4.62**	**7.176**	**0.001**	**0.070**	**4.58**	**4**	**4.81**	**423**	**8.702**	**0.000**	**0.084**
	Number of Additions	**2.33**	**2.75**	**2.61**	**3.35**	**3.828**	**0.023**	**0.039**	**1.91**	**2.24**	**2.46**	**2.93**	**4.768**	**0.010**	**0.048**
	Number of Omissions	**2.72**	**2.57**	**2.96**	**2.97**	**3.858**	**0.023**	**0.039**	**2.94**	**4.01**	**3.31**	**3.01**	**6.312**	**0.002**	**0.062**
	Number of Inversions	0.17	0.52	0.19	0.53	0.121	0.886	0.001	0.20	1.15	0.22	0.79	0.160	0.853	0.002
	Number of Rotations	–	–	–	–	.	.	.	0.01	0.11	0.04	0.47	0.825	0.440	0.009
	Number of Lexicalizations	0.12	0.46	0.08	0.33	0.474	0.623	0.005	0.16	0.54	0.06	0.28	2.206	0.113	0.023
	Number of incorrect accents	7.95	5.25	8.43	5.13	0.239	0.787	0.002	8	4.84	8.55	4.75	1.531	0.219	0.016
	TOTAL	**17.95**	**10.99**	**18.98**	**12.88**	**3.407**	**0.035**	**0.034**	**17.79**	**12.66**	**19.46**	**12.27**	**6.633**	**0.002**	**0.065**
GRAMMAR	Number of grammatically incorrect sentences	4.57	3.04	4.55	2.99	0.279	0.757	0.003	**4.31**	**2.85**	**4.50**	**2.88**	**4.682**	**0.010**	**0.047**
MAIN AND SECONDARY IDEAS	Number of main ideas	1.11	0.42	1.14	0.40	2.286	0.104	0.023	1.26	0.57	1.23	0.48	0.730	0.483	0.008
	Number of secondary ideas	**9.80**	**3.96**	**10.27**	**5.09**	**4.528**	**0.012**	**0.045**	9.33	3.94	9.88	4.40	2.657	0.073	0.027
PLANNING ERRORS	Number of disconnections between the main idea and the title	0.40	0.75	0.44	0.72	1.408	0.247	0.015	**0.74**	**1.18**	**0.41**	**0.84**	**3.313**	**0.039**	**0.034**
	Number of times that secondary ideas do not appear	0.06	0.46	0	0	1.216	0.299	0.013	0.06	0.40	0.02	0.19	1.308	0.273	0.014
	Number of deviations from thematic continuity	0.20	0.46	0.23	0.50	1.041	0.355	0.011	0.31	0.66	0.22	0.48	1.689	0.187	0.017
	Number of non-uses of technical vocabulary	1.57	1.78	1.32	1.73	0.529	0.590	0.006	**1.62**	**1.79**	**0.98**	**1.30**	**6.468**	**0.002**	**0.063**
	Number of non-uses of coherent vocabulary	0.07	0.49	0.04	0.39	0.857	0.426	0.009	0.12	0.75	0.03	0.28	1.530	0.219	0.016
	Number of non-uses of varied vocabulary	1.80	1.74	1.48	1.61	1.042	0.355	0.011	1.65	1.87	1.18	1.43	2.020	0.136	0.021
	TOTAL	4.10	4.47	3.51	3.62	1.014	0.365	0.011	**4.51**	**5.07**	**2.83**	**3.05**	**5.379**	**0.005**	**0.053**
VOCABULARY	Number of uses of technical vocabulary	**9.19**	**6.51**	**10.31**	**6.07**	**7.421**	**0.001**	**0.072**	**11.02**	**7.72**	**11.78**	**6.72**	**9.929**	**0.000**	**0.094**
	Number of uses of coherent vocabulary	0.06	0.40	0.04	0.47	1.120	0.329	0.012	0.14	0.89	0.16	1.26	.873	0.419	0.009
	Number of uses of varied vocabulary	**3.04**	**2.32**	**3.61**	**2.74**	**7.882**	**0.001**	**0.076**	**3.74**	**2.61**	**3.69**	**2.71**	**7.553**	**0.001**	**0.073**
	TOTAL	**12.28**	**8.23**	**13.96**	**8.19**	**8.447**	**0.000**	**0.081**	**14.90**	**9.60**	**15.63**	**8.57**	**10.240**	**0.000**	**0.097**
REVISION	Number of times modifications were made to the text	**0.46**	**0.84**	**0.82**	**1.21**	**6.616**	**0.002**	**0.065**	0.58	0.84	0.90	1.11	2.805	0.063	0.029
**NET TOTAL**		**133.95**	**70.59**	**152.14**	**92.68**	**15.48**	**0.000**	**0.140**	**128.15**	**74**	**143.59**	**75.38**	**13.918**	**0.000**	**0.127**

Bold values in the tables represent those values that are statistically significant (p ≤ 0,05).

For the dependent variables of the category *Errors Related to Formal Aspects* (Number of punctuation errors, number of lines not respecting the margins, number of incorrectly separated words, number of incorrect conjunctions between words, number of repetitions, number of words with unreadable handwriting, and Total) the ANCOVAs revealed statistically significant differences in the number of incorrect conjunctions between words (F_2,191_ = 4.5558; Mce =15.15; p<.05) with scores being higher for the OCPD group than the ASPD group, and Total score (F_2,191_ = 3.723; Mce =185.94; p<.05) with scores being higher for the OCPD group than the ASPD group. No statistically significant differences were found in the number of punctuation errors, the number of lines not respecting the margins, incorrectly separated words, repetitions, and the number of words with unreadable handwriting.

For the dependent variables of the *Decoding Errors* category (Number of substitutions, number of additions, number of omissions, number of inversions, number of rotations, number of lexicalizations, number of incorrect accents, and Total), the ANCOVAs revealed statistically significant differences in the number of substitutions (F_2,191_ = 7.176; Mce =122.937; p<.01) with higher scores for the OCPD group than the ASPD group; number of additions (F_2,191_ = 3.828; Mce =35.848; p<.05) with the OCPD group obtaining higher scores than the ASPD group; number of omissions (F_2,191_ = 3.858; Mce =29.494; p<.05), with the OCPD group scoring higher than the ASPD group; and Total score (F_2,191_ = 3.407; Mce =486.908; p<.05), with scores being higher for the OCPD group than the ASPD group. No statistically significant differences were found in the number of inversions, number of rotations, number of lexicalizations, and number of incorrect accents.

For the dependent variables of the Categories *Grammar* (Number of grammatically incorrect sentences); *Revision* (Number of modifications made to the text), and *Net Total*, ANCOVAs revealed statistically significant differences in *Revision*: Number of modifications made to the text (F_2,191_ = 6.616; Mce =7.349; p<.01) with scores being higher for the OCPD group than the ASPD group and *Net Total* (F_2,191_ = 15.482; Mce =95989.637; p<.001) with scores being higher for the OCPD group than the ASPD group. No statistically significant differences were found in *Grammar* (Number of grammatically incorrect sentences).

For the dependent variables of the *Main and Secondary Idea* **s** category (Number of main ideas and Number of secondary ideas), ANCOVAs revealed statistically significant differences in the number of secondary ideas (F_2,191_ = 4.528; Mce =94.266; p<.05) with higher scores for the OCPD group than the ASPD group. No statistically significant differences were found in the number of main ideas.

For the dependent variables of the *Vocabulary* Category (Number of uses of technical vocabulary; Number of uses of consistent vocabulary; Number of uses of varied vocabulary, and Total score), the ANCOVAs revealed statistically significant differences in the number of uses of technical vocabulary (F_2,191_ = 7.421; Mce =272.905; p<.01) with scores being higher for the OCPD group than the ASPD group; Number of varied vocabulary uses (F_2,191_ = 7.882; Mce =48.921; p<.01) with scores being higher for the OCPD group than the ASPD group, and Total score (F_2,191_ = 8.447; Mce =530.953; p<.001) with scores being higher for the OCPD group than the ASPD group. However, no statistically significant differences were found in a number of uses of coherent vocabulary (see **Table 2**).

### Analysis of essays categorization

The MANCOVAS revealed statistically significant results for the Categories *Words and Paragraphs* (Number of words and Number of paragraphs; Wilks’ Lambda = 0.873, F_2,190_ = 13.779; p <.001); *Errors Related to Formal Aspects* (Number of punctuation errors, number of lines not respecting the margins, number of incorrect separations between words, number of incorrect conjunctions between words, number of repetitions, number of words with unreadable handwriting, and Total; Wilks’ Lambda = 0.677, F_6,186_ = 14.794; p <.001); *Decoding Errors* (Number of substitutions, number of additions, number of omissions, number of inversions, number of rotations, number of lexicalizations, number of incorrect accents, and Total; Wilks’ Lambda = 0.625, F_8,184_ = 13.804; p <.001); *Grammar* (Number of grammatically incorrect sentences); *Revision* (Number of modifications made to the text) and *Net Total*; Wilks’ Lambda = 0.69, F_3,189_ = 28.28; p <.001); *Main and Secondary Ideas* (Number of main ideas and Number of secondary ideas; Wilks’ Lambda = 0.661, F_2,190_ = 48.650; p <.001); *Planning Errors* (Number of disconnections between main idea and title, number of times secondary ideas do not appear, number of deviations from thematic continuity, number of times technical vocabulary not used, number of times coherent vocabulary not used, number of times varied vocabulary not used, and Total; Wilks’ Lambda = 0.841, F_6,186_ = 5.855; p <.001); *Vocabulary* (Number of uses of technical vocabulary, number of uses of coherent vocabulary, number of uses of varied vocabulary, and Total; Wilks’ Lambda = 0.95, F_3,189_ = 3.33; p <.05).

Univariate ANCOVAs conducted for each of the levels of the dependent variables of the *Words and Paragraphs* Category (Number of Words and Number of Paragraphs) revealed statistically significant differences in number of words (F_2,191_ = 6.630; Mce =29779.342; p<.01) with higher scores for the OCPD group than the ASPD group; in Number of paragraphs (F_2,191_ = 12.476; Mce =13.971; p<.001) with the OCPD group obtaining higher scores than the ASPD group (See **Table 2**).

For the dependent variables of the Category *Errors Related to Formal Aspects* (Number of punctuation errors, number of lines not respecting margins, number of incorrect separations between words, number of incorrect conjunctions between words, number of repetitions, number of words with unreadable handwriting and Total) the ANCOVAs revealed statistically significant differences in number of punctuation errors (F_2,191_ = 3.337; Mce =74.399; p<.05) with scores being higher for the OCPD group than the ASPD group, and Total score (F_2,191_ = 6.238; Mce =253.868; p<.01) with scores being higher for the OCPD group than the ASPD group. No statistically significant differences were found in the number of lines that did not respect the margins; number of incorrect separations between words; number of incorrect conjunctions between words; number of repetitions, and number of words with unreadable handwriting.

For the dependent variables of the *Decoding Errors* category (Number of substitutions, number of additions, number of Omissions, number of inversions, number of rotations, number of lexicalizations, number of incorrect tildes, and Total), the ANCOVAs revealed statistically significant differences in number of substitutions (F_2,191_ = 8.702; Mce =137.266; p<.001), showing higher scores for the OCPD group than the ASPD group; number of additions (F_2,191_ = 4.768; Mce =32.777; p<.05) with scores being higher for the OCPD group than the ASPD group; number of omissions (F_2,191_ = 6.312; Mce =71.529; p<.01) with the OCPD group showing higher scores than the ASPD group, and Total score (F_2,191_ = 6.633; Mce =968.669; p<.01), with the OCPD group obtaining higher scores than the ASPD group. However, no statistically significant differences were found in the number of inversions, number of rotations, number of lexicalizations, and number of incorrect accents.

For the dependent variables of the Categories *Grammar* (Number of grammatically incorrect sentences); *Revision* (Number of modifications made to the text), and *Net Total*, ANCOVAs revealed statistically significant differences in Grammar (Number of grammatically incorrect sentences) (F_2,191_ = 4.682; Mce =39.904; p<.05) with scores being higher for the OCPD group than the ASPD group, and Net Total (F_2,191_ = 13.918; Mce =69059.771; p<.001) with scores being higher for the OCPD group than the ASPD group. No statistically significant differences were found in Revision (Number of modifications made to the text).

For the dependent variables of the *Planning Errors* Category (Number of disconnections between the main idea and the title, number of times secondary ideas do not appear, number of deviations from thematic continuity, number of times technical vocabulary not used, number of times coherent vocabulary not used, number of times varied vocabulary not used, and Total) the ANCOVAs revealed statistically significant differences in the number of disconnections between the main idea and the title (F_2,191_ = 3.313; Mce =3.287; p<.05) with the scores being higher for the ASPD group than he OCPD group; number of times technical vocabulary not used; (F_2,191_ = 6.468; Mce =14.783; p<.01) with scores being higher for the ASPD group than the OCPD group; and Total score (F_2,191_ = 5.379; Mce =86.235; p<.01) with the ASPD group obtaining higher scores than the OCPD group. No statistically significant differences were found in the number of times secondary ideas did not appear, the number of deviations from thematic continuity, the number of times coherent vocabulary was not used, and the number of times varied vocabulary was not used.

For the dependent variables of the *Vocabulary* Category (Number of uses of technical vocabulary; Number of uses of coherent vocabulary; Number of uses of varied vocabulary, and Total score), the ANCOVAs revealed statistically significant differences in the number of uses of technical vocabulary (F_2,191_ = 9.929; Mce =464.199; p<.001) with scores being higher for the OCPD group than the ASPD group; Number of varied vocabulary uses (F_2,191_ = 7.553; Mce =50.197; p<.01) with scores being higher for the ASPD group than the OCPD group, and Total score (F_2,191_ = 10.24; Mce =756.371; p<.001) with scores being higher for the OCPD group than the ASPD group. No statistically significant differences were found in a number of uses of coherent vocabulary.

For the dependent variables of the *Main and Secondary Ideas* Category (Number of main ideas and number of secondary ideas), the ANCOVAs did not reveal statistically significant differences (See **Table 2**).

## Discussion

This study aimed to analyze the profiles in terms of the proposed coding from the PROESC in terms of personality disorders (ASPD/DPCC and OCPD/GVC) in the prison population.

In the writing of narratives and essays, we found statistically significant group differences in the *Number of Words and Number of Paragraphs*, with the OCPD/GVC group obtaining higher scores than the ASPD/DPCC group. This is a novel finding and could be explained by the fact that the narratives and essays are informal and unstructured tasks (they received few instructions, were freely themed, and participants were given the time they needed). Individuals with OCPD/GVC can create longer texts because of the absence of a time limit and because they use more frequent words with which they feel they have more control and fluency, which is characteristic of this personality profile (26).

Regarding the *Errors Related to Formal Aspects*, the scores were higher for the OCPD/GVC group than the ASPD/DPCC group in both narratives and essays. Specifically, in the short narratives, the OCPD/GVC group scored higher on the Number of incorrect word conjunctions and Total score than the ASPD/DPCC group. For the essays, the OCPD group showed higher scores on the number of punctuation errors and Total score than the ASPD/DPCC group. This means that the OCPD/GVC group made more errors than the ASPD/DPCC group in the analyzed variables related to grammar and phonological awareness. However, the OCPD/GVC group compensates and avoids errors by showing above average performance on other skills, such as memory or known vocabulary (12).

Concerning *Decoding Errors*, we found that for both narratives and essays, the OCPD/GVC group performed better than the ASPD/DPCC group. Specifically, in the narratives, we found statistically significant differences in performance regarding number of substitutions, number of additions, number of omissions, and total scores, all of which were higher for the OCPD/GVC group than the ASPD/DPCC group. For the essays, we found significant differences in the number of substitutions, number of additions, number of omissions, and total score, being, all of which were higher for the OCPD/GVC group than the ASPD/DPCC group. These results are novel and agree with those obtained in other studies (12, 19, 27, 28) showing that individuals with dyslexia present great difficulties in using basic spelling rules. However, another study found that participants with dyslexia did not make more decoding errors than control or non-dyslexic participants (29).

Regarding the categories *Grammar, Revision*, and *Net Total*, we found that the OCPD/GVC group obtained higher scores on grammar in the essays than the ASPD/DPCC group; that is, they have shown more difficulties in this category. In addition, the OCPD/GVC group obtained higher net total scores on essays and narratives than the ASPD/DPCC group, which indicates that they have more difficulties in general. However, for the narratives, the OCPD/GVC group performed better on Revision than the ASPD/DPCC group. These findings could be explained by the fact that the dyslexia profile is characterized by showing difficulties in grammar and syntax (16, 18). In addition, the net total scores obtained by OCPD/GVC group are particularly noteworthy, since these indicate writing difficulties similar to those observed in dyslexia. However, in the Revision category, the OCPD/GVC group performed better on the task than the ASPD/DPCC group. This category is intuitive (placing higher demands on cognitive processes such as attention, memory, and concentration) and the errors are related to analysis and error detection. We can highlight, therefore, the characteristic OCPD/GVC profile that includes preoccupation with details, rules, lists, order, and perfectionism (26).

Concerning the *Main and Secondary Ideas* Category, we also found that the OCPD/GVC group scored higher than the ASPD/DPCC group in the number of secondary ideas in the narratives. For example, in the *Planning Errors* Category, we found statistically significant differences in the number of disconnections between the main idea and the title, the number of times that technical vocabulary was not used, and the Total score in the essays, all of which were the higher for the ASPD/DPCC group than the OCPD/GVC group. Although contrary to the findings observed in the rest of the categories, these results are quite novel. Although we know that a typical error observed in dyslexia is the difficulty or problems in creating sequences and ordering them, together with the enrichment of such content (30), we can again, highlight the OCPD/GVC profile, which is characterized by a preoccupation with organization, planning and excessive dedication to work and productivity. These tasks focus on mental processes such as planning that include operations such as idea generation and organization to create a design of what is written and how it will be written (18).

And finally, concerning the *Vocabulary* Category, we found statistically significant differences in the narratives regarding the number of uses of technical vocabulary, number of uses of varied vocabulary, and total score, all of which were higher for the OCPD/GVC group than the ASPD/DPCC group. In a similar vein, when analyzing the essays, in this same category (Vocabulary**),** we found statistically significant differences in the number of uses of technical vocabulary and total score, which, in both cases, were higher for the OCPD/GVC group than the ASPD/DPCC group. On the other hand, the ASPD/DPCC group obtained higher scores than the OCPD/GVC group on the number of varied vocabulary uses. This result, although contradictory, is in line with what has been described previously regarding the characteristic profile of OCPD/GVC, including preoccupation with details, rules, lists, order, organization, or schedules to the point of losing sight of the main object of the activity. Moreover, they are perfectionists and even tend to enrich their vocabulary (characteristic of dyslexia). Thus, OCPD/GVC is better in all categories because it uses more appropriate vocabulary, although it takes time to access long-term memory (31).

On the contrary, our observation that varied vocabulary use in the essays was higher in the ASPD/DPCC group than the OCPD/GVC group could be due to possible impairments in the working memory of individuals with OCPD/GVC. According to the literature (30, 32), these deficits could explain the presence of dyslexia in OCPD/GVC; that is, they do not have adequate access to general vocabulary in terms of both variety and richness. However, for individuals with OCPD/GVC, the retrieval of technical vocabulary would be more precise when these are frequently used words.

Currently, treatments aimed at the prison population present several problems. These problems could be due to the lack of specificity of the content of the treatment, which result from the lack of knowledge of OCPD/GVC and its relationship with dyslexia (31). This lack of knowledge could underlie the ineffectiveness of interventions for reintegrating the prison population. Therefore, knowledge of language problems in this population in general and in OCPD/GVC in particular could even help to reduce recidivism and improve the effectiveness of targeted interventions in the prison population.

Our results are consistent with multiple studies on text writing (16, 18). The elevated scores in the text writing task of OCPD/GVC participants could be due to the profile characteristics mentioned above, since these tasks involve the use of analysis, error detection, organization, and planning tools. One significant aspect of this text writing task that could negatively affect ASPD/DPCC scores is the profile that characterizes them (lack of self-control, planning and attention problems, and irresponsibility in task execution) (18).

In summary, our study has yielded three main findings. First, the OCPD/GVC group scored better than the ASPD/DPCC group on the categories of number of words and number of paragraphs in narratives and essays, errors related to formal aspects of narratives, errors in decoding narratives, revision and the net total score on narratives, main and secondary ideas of narratives, the vocabulary of narratives, errors related to formal aspects of essays, errors in decoding essays, and grammar and the net total score on essays. Second, the OCPD/GVC and ASPD/DPCC groups did not differ on tasks related to narrative grammar, narrative planning errors, or revision and main and secondary ideas of the essays. Third, ASPDs/DPCCs scored better than OCPDs/GVC on the categories of errors in planning and vocabulary of essays (number of varied vocabulary uses). This difference is in accord with the results of other studies (16, 18, 31) that have reported how some aspects such as planning directly influence text production, and it is these aspects that are the most difficult to learn and acquire. Moreover, if we consider the predisposition to develop dyslexia and social exclusion factors, we can explain the discrepant results found in this study. According to these same studies (16, 18, 31), youngsters who have not acquired writing skills during school are very likely to fail to write correctly. Moreover, recent studies (16, 29, 32) have shown that many students have not acquired the necessary skills to develop writing correctly. These problems in phonological awareness and phoneme-grapheme correspondence may prevail until adulthood. In addition, and in a similar vein, other studies (31) have added that writing automation takes place between elementary school (6-11 years) and the age of 15 years. Although the associated alterations may be readily treatable, it is necessary to detect such difficulties. If these problems are added to a potential disorder in reading and writing, such as dyslexia, together with school dropout, we could find a common profile characterized by OCPD/GVC, dyslexia, and social exclusion.

As with all research, our results must be evaluated in the context of several limitations. The main limitation is the absence of a non-custodial control group and a non-custodial dyslexia group, with which we should have compared the results we have presented. In addition, the language difficulties explored in this study require a more exhaustive analysis of the cognitive processes involved, such as learning, attention, working memory, and executive functions. Besides, the sample analyzed only included men. However, this was the case for the following three reasons: 1) one of the crimes analyzed was gender violence, which is understood as male aggression toward women; 2) no women were serving a prison sentence for intimate partner violence; and 3) the prison population contains five times more men than women, so that, given our inclusion and exclusion criteria, it would have been impossible to conduct this study with women. However, no other study has provided a separate in-depth analysis of each component of these writing tasks.

This study is novel since it focuses on aspects that have scarcely been studied in the literature, such as the relationship between personality disorders and language difficulties and the analysis of linguistic differences between OCPD/GVC and ASPD/DPCC. Our results confirm the need for speech-language pathology and therapy intervention in the prison population. Such an intervention could have considerable legal, social, and economic impact. In particular, a multidisciplinary intervention program that includes speech-language therapy would exponentially enrich prison care and education. Such a program could detect, in an early manner, those cases where we find a negative education outcome, a language difficulty, or a personality disorder (OCPD/GVC) that would otherwise lead to aggressive and maladaptive behaviors. However, such a program would not only focus on preventive aspects in the penitentiary environment but would also include social and labor reinsertion. Improving language enriches the possibilities of finding employment and communicating more effectively with the environment, both immediate and far. In addition, the improvement of language could favor the conscience of the prisoner and regenerate the negative vision that society has of this population.

We believe that in the future, other disorders such as attention deficit hyperactivity disorder (ADHD), dyslexia, or other difficulties should be evaluated in the prison population. In addition, Electroencephalogram (EEG) and functional Magnetic Resonance Imaging (fMRI) should be employed to reveal common neuropsychological mechanisms underlying compulsivity and language pathologies that may affect vulnerability to gender violence in particular or criminal behavior in general. In addition, a further element that could enrich other similar studies is the use of techniques that measure eye movements. Although these are very complex measures, they can be very useful when information cannot be obtained by other simpler means and when some aspect of the response is related to the variable under study.

## Conclusions

Although individuals know phoneme-grapheme correspondence rules, language disturbances of a reiterative and persistent nature may appear in those who show compulsive behavior. This finding could be related to co-occurrences in the behavior of compulsive individuals and those with learning difficulties. Language therapy in patients with high levels of compulsivity could improve self-control and self-criticism, thereby enhancing the capacity to form social relationships and show empathy.

## Data availability statement

The datasets presented in this study can be found in online repositories. The names of the repository/repositories and accession number(s) can be found below: http://dx.doi.org/10.30827/Digibug.89489.

## Ethics statement

The studies involving humans were approved by Comité Ético de Consejería de Salud y Familia, Junta de Andalucia, España (PEIBA, 0766-N-21). The studies were conducted in accordance with the local legislation and institutional requirements. The participants provided their written informed consent to participate in this study.

## Author contributions

LM-L: Conceptualization, Data curation, Formal analysis, Funding acquisition, Investigation, Methodology, Project administration, Resources, Software, Supervision, Validation, Visualization, Writing – original draft, Writing – review & editing. B-F-G-V: Methodology, Resources, Writing – review & editing, Investigation, Supervision, Visualization. SL-R: Methodology, Writing – review & editing, Conceptualization, Writing – original draft. MS-B: Methodology, Writing – review & editing, Resources.
